# Unveiling the non-linear synergistic effects of smoking and aging on cataract-AMD comorbidity: an explainable artificial intelligence approach

**DOI:** 10.3389/fpubh.2026.1784189

**Published:** 2026-04-13

**Authors:** Mingxia Tang, Yu Liu, Lili Dong

**Affiliations:** Department of Ophthalmology, The Affiliated Taizhou People's Hospital of Nanjing Medical University, Taizhou School of Clinical Medicine, Nanjing Medical University, Taizhou, Jiangsu, China

**Keywords:** Cataract and AMD comorbidity, Explainable AI (XAI), machine learning, risk stratification, XGBoost

## Abstract

**Background:**

The comorbidity of cataract and age-related macular degeneration (AMD) poses a significant public health burden. Traditional linear statistical models often fail to capture complex, non-linear interactions among risk factors. This study aimed to develop an interpretable machine learning framework to predict comorbidity risk and elucidate the synergistic effects of systemic and ocular factors.

**Methods:**

A retrospective case-control study was conducted involving 640 participants (264 comorbidity cases and 376 controls). Fifteen multi-dimensional clinical features were extracted. Four machine learning algorithms—Logistic Regression, Random Forest, SVM, and XGBoost—were trained and validated. Model performance was assessed via AUROC, AUPRC, and calibration curves. SHapley Additive exPlanations (SHAP) and LIME were employed to visualize global and local interpretability.

**Results:**

The XGBoost model demonstrated robust discriminative performance (AUC = 0.895, 95% CI: 0.85–0.93) and calibration compared to other algorithms. SHAP analysis identified drusen severity and lens opacity (LOCS III) as dominant ocular predictors, while C-reactive protein (CRP) and smoking were critical systemic contributors. Notably, interaction analysis revealed a non-linear synergistic effect: smoking was associated with an exponentially higher comorbidity risk in individuals aged >75 years, whereas CRP exhibited a distinct saturation threshold effect. Decision curve analysis confirmed the model's high net clinical benefit across a wide range of threshold probabilities.

**Conclusion:**

This study establishes a robust, clinically applicable risk stratification tool for cataract and AMD comorbidity. By uncovering non-linear interactions between aging, lifestyle, and inflammation, it provides valuable evidence-based support for personalized screening and preventive intervention.

## 1 Introduction

The two leading causes of visual impairment and irreversible blindness globally, particularly among the geriatric population, are cataract and age-related macular degeneration (AMD) (1–3). With the rapid aging of the global population, the co-occurrence of these two conditions—termed comorbidity—has emerged as a critical public health issue, imposing a significant burden on healthcare systems and severely compromising patients' quality of life (4, 5). Clinically, the presence of dense cataracts often obscures the fundus, delaying the timely diagnosis of underlying AMD (6). Furthermore, cataract extraction surgery in patients with pre-existing AMD requires cautious preoperative evaluation due to potential inflammatory responses that may exacerbate macular degeneration (7). Therefore, identifying patients at high risk for this comorbidity prior to surgical intervention is of paramount clinical importance. There are epidemiological indications that cataract and AMD are related in their pathogenic pathways, such as oxidative stress, chronic systemic inflammation, and metabolic dysregulation (8). Moreover, the common shared risk factors, including advanced age, smoking, and hypertension, have consistently been implicated in the progression of both diseases (9, 10).

Nonetheless, conventional epidemiological research has largely utilized linear statistical analysis, including logistic regression, in determining risk factors (11, 12). For instance, previous population-based studies have successfully established independent associations between smoking and AMD progression using standard regression models (13, 14). However, these traditional methods operate under the strict assumption of linearity and variable independence. They often fail to capture complex, non-linear, and high-dimensional interactions inherent in biological systems. Specifically, existing models may overlook the specific synergistic action of lifestyle factors (e.g., smoking) and physiological aging mechanisms, leading to an underestimation of risk among certain high-risk subgroups (15). Furthermore, while recent deep learning studies focus heavily on image-based diagnosis, there is a gap in integrating structured clinical data with interpretable algorithms to reveal systemic pathogenic mechanisms (16).

Over the past few years, machine learning (ML) algorithms, especially ensemble algorithm such as Extreme Gradient Boosting (XGBoost), have proven to be more useful in the analysis of complex clinical data and discovery algorithmic patterns that are non-linear (17–20). Nevertheless, the clinical implementation of ML models is frequently prevented by their black-box character, which fails to bring transparency in the decision-making process (21). On top of such high predictive accuracy, Explainable Artificial Intelligence (XAI) methods have been proposed to facilitate the bridging of high predictive accuracy with clinical interpretability (22, 23). By unlocking the algorithmic “black box,” XAI provides intuitive visual evidence of how individual risk factors interact and contribute to disease probability, which is essential for fostering physician trust and facilitating its adoption in routine clinical practice (24).

Thus, the main contributions of this research are:

1. The development of a robust XGBoost-based framework to predict cataract and AMD comorbidity using multi-dimensional clinical data, involving the evaluation of multiple machine learning algorithms and the ultimate deployment of an XGBoost-based model.
2. The application of XAI (SHAP and LIME) to elucidate complex, non-linear synergistic effects, specifically the interaction between aging and smoking.
3. The creation of a practical, web-based risk calculator to facilitate personalized clinical screening and decision-making.

## 2 Methods

### 2.1 Study design and participant selection

**Figure 1:**
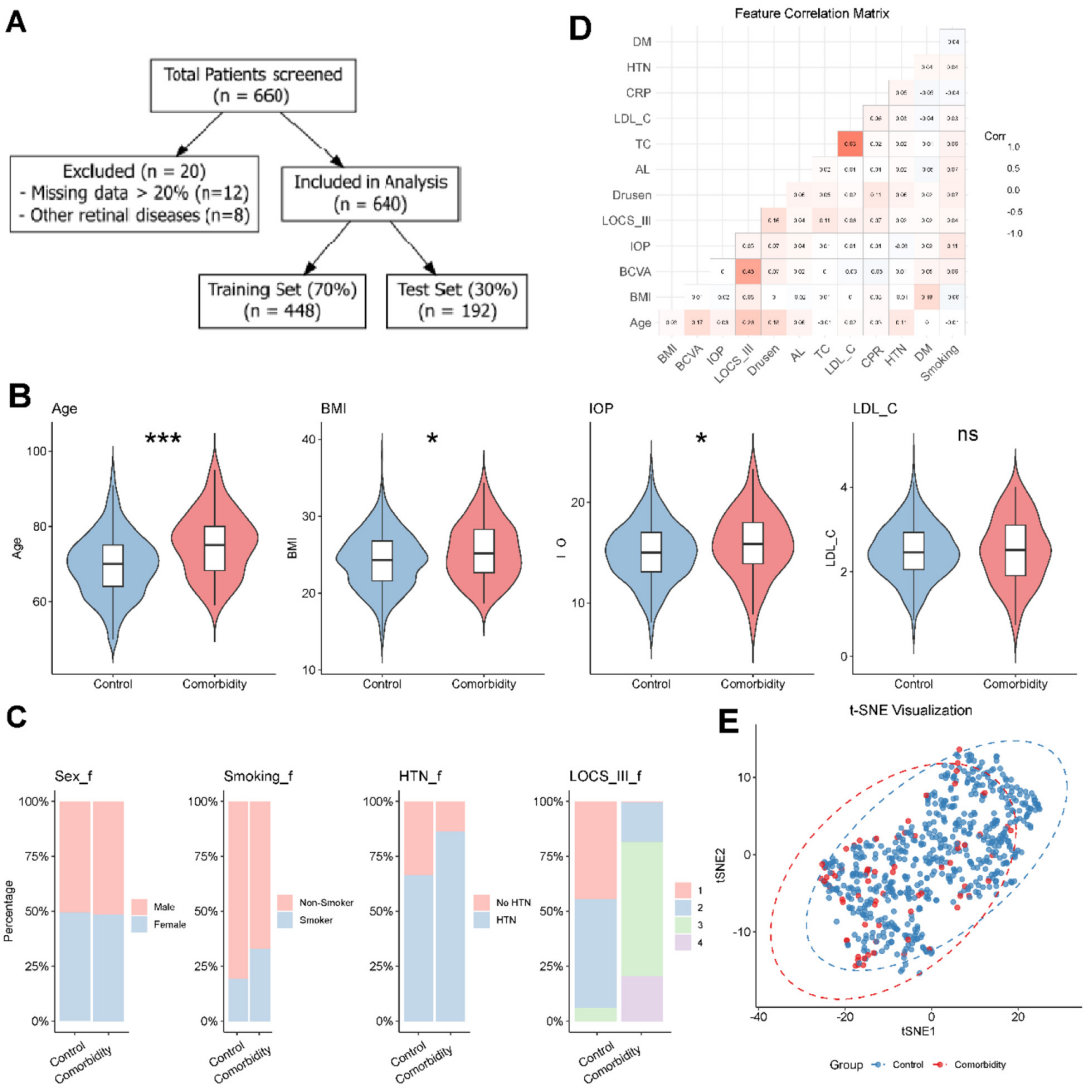
Study workflow and baseline characteristics of the study population. **(A)** Flowchart illustrating the patient selection process, including inclusion/exclusion criteria and dataset splitting (training set 70%, test set 30%). **(B)** Violin plots comparing the distribution of continuous variables (Age, BMI, IOP, LDL-C) between the control and comorbidity groups. The internal box plots represent the median and interquartile ranges. Asterisks indicate statistical significance (*P < 0.05; **P < 0.01; ***P < 0.001; ns, not significant). **(C)** Stacked bar charts displaying the proportions of categorical variables [Sex, Smoking Status, Hypertension (HTN), and Lens Opacities Classification System III (LOCS III)] across groups. **(D)** Correlation heatmap (Spearman's rank correlation) of the 15 clinical features included in the analysis. **(E)** t-Distributed Stochastic Neighbor Embedding (t-SNE) visualization of the high-dimensional data, showing the distribution and separability of the control (blue) and comorbidity (red) groups in the feature space.

This retrospective case-control study was conducted to develop a predictive model for the comorbidity of cataract and age-related macular degeneration (AMD). The study protocol adhered to the tenets of the Declaration of Helsinki and was approved by the Institutional Review Board (IRB) of the Affiliated Taizhou People's Hospital of Nanjing Medical University (Approval No. KL901014). Given the retrospective nature of the study design and the utilization of strictly de-identified datasets, the requirement for written informed consent was waived by the Institutional Review Board. The study workflow is illustrated in Figure 1A. A total of 660 individuals were initially screened from the hospital electronic medical record system between January 2021 and June 2024. The inclusion criteria comprised patients aged 50 years or older with complete ophthalmic and systemic examination records. Exclusion criteria were defined as follows: (1) presence of other confounding retinal pathologies (e.g., diabetic retinopathy, retinal vein occlusion) (*n* = 8); and (2) incomplete clinical data exceeding 20% missingness per subject (*n* = 12). Ultimately, 640 participants were included in the final analysis and were randomly partitioned into a training set (70%, *n* = 448) and an independent test set (30%, *n* = 192) using stratified sampling to preserve the proportion of outcome classes.

### 2.2 Data collection and variable definition

We extracted 15 multi-dimensional clinical indicators categorized into three domains: demographic, ophthalmic, and systemic domains, to form a holistic risk profile of each participant. Demographic and lifestyle variables incorporated the age, gender, body mass index (BMI), smoking habits and alcohol history. The ophthalmic parameters included the best-corrected visual acuity [BCVA (transformed into logMAR))] intraocular pressure (IOP), axial length (AL), the severity of lens opacities (scores 0–3) based on the Lens Opacities Classification System III, and the severity of drusen (graded 0–3). Also, systemic and metabolic biomarkers were also considered and included history of hypertension (HTN), diabetes mellitus (DM), total cholesterol (TC), low-density lipoprotein cholesterol (LDL-C) and C-reactive protein (CRP) levels. The main outcome was formulated as comorbidity of cataract and AMD that was established using normal fundus photography and Optical Coherence Tomography (OCT) image examined by qualified ophthalmologists.

### 2.3 Data preprocessing and exploration

To avoid the scale inequalities, the z-score normalization of the continuous variables was used before modeling. Categorical variables have been one-hot coded. To visualize the underlying data structure and assess feature separability between the control and comorbidity groups, t-Distributed Stochastic Neighbor Embedding (t-SNE) was employed. Any gaps in included variables (with < 20% missing values) were filled with k-Nearest Neighbors algorithm (k-NN).

### 2.4 Machine learning model development

Four supervised machine learning algorithms were developed to form the risk prediction models, including Logistic Regression (LR), Random Forest (RF), Support Vector Machine (SVM), and Extreme Gradient Boosting (XGBoost). Given the inclusion of 15 clinical features and 264 positive comorbidity events in our total cohort of 640 participants, our dataset comfortably exceeded the recommended Events Per Variable (EPV) threshold of 10, ensuring adequate statistical power for machine learning applications. The 10-fold cross-validation on the training set was applied to optimize the performance of the model and avoid overfitting. Hyperparameter tuning was conducted via an exhaustive grid search. For the primary XGBoost model, the optimal parameters were determined as follows: learning rate (eta) = 0.05, maximum tree depth (max_depth) = 4, and number of boosting rounds (nrounds) = 150, utilizing a binary logistic objective function. The XGBoost model, known for its efficiency in handling non-linear relationships and resilience to overfitting on medium-sized tabular datasets, was prioritized for detailed feature interaction analysis.

### 2.5 Model evaluation and statistical analysis

To assess the model discrimination, the Receiver Operating Characteristic (ROC) curve and Area Under the Curve (AUC) were employed. Since the effect of imbalance in the classes may exist, the Precision-Recall Curves (PRC) and the Area Under the PRC (AUPRC) were also determined. Radar charts were used to depict the overall performance metrics as accuracy, sensitivity, specificity, positive predictive value (PPV), negative predictive value (NPV), and F1-score. To determine how predicted probabilities were in agreement with observed frequencies, the Brier score was used to plot calibration curves.

In order to measure clinical utility, Decision Curve Analysis (DCA) and Clinical Impact Curves (CIC) were created to measure the net benefit of the model at a range of different threshold probabilities in contrast to the strategies of treat-all and treat-none.

The entire statistical testing was conducted through the use of R software (version 4.3.1). The Student *t*-test or Mann-Whitney U test was applied to compare the continuous variables whereas Chi-square test was used to compare categorical variables. The *P*-value of less than 0.05 was regarded as significant.

### 2.6 Interpretability framework

To address the “black-box” nature of machine learning, we integrated global and local interpretability techniques. SHapley Additive exPlanations (SHAP) based on cooperative game theory were used to quantify feature importance, directional effects (Beeswarm plots), and non-linear dependencies. The interpretation of SHAP values followed specific steps: first, the absolute SHAP values were averaged to rank global feature importance; second, the directionality was assessed, where SHAP values greater than zero indicated an increased risk of comorbidity, and values below zero indicated a protective effect. In summary plots, color coding (red for high feature values, blue for low) was utilized to determine how the magnitude of a clinical variable correlated with disease risk. SHAP interaction values were calculated to explore synergistic effects between risk factors (e.g., Age and Smoking). For individual-level explanation, SHAP waterfall plots and Local Interpretable Model-agnostic Explanations (LIME) were employed to visualize feature contributions for specific high-risk and low-risk cases.

## 3 Results

### 3.1 Study population characteristics and data exploration

The study group enrolled a total of 640 participants who were chosen out of an original number of 660 screened participants after the elimination of 20 patients because of the absence of data or comorbid retinal pathologies (Table 1 and Figure 1A). A random split of the dataset was done into a training set (70% *n* = 448) and a test set (30% *n* = 192).

**Table 1:** Baseline demographic and clinical characteristics of the study population.

Characteristics	Control group (*n* = 376)	Comorbidity group (*n* = 264)	*P*-value
Demographic and lifestyle factors			
Age, years (mean ± SD)	67.7 ± 7.9	73.8 ± 8.3	**< 0.001**
Sex, male, no. (%)	190 (50.5%)	136 (51.5%)	0.869
Body mass index, kg/m^2^ [median (IQR)]	24.1 [21.8, 25.9]	25.3 [21.5, 28.4]	**< 0.05**
Smoking history, yes, no. (%)	72 (19.1%)	87 (33.0%)	**< 0.001**
Alcohol consumption, yes, no. (%)	112 (29.8%)	55 (20.8%)	**0.014**
Ophthalmic parameters			
BCVA, logMAR [median (IQR)]	0.46 [0.35, 0.58]	0.85 [0.75, 0.95]	**< 0.001**
Intraocular pressure, mmHg (median [IQR])	14.9 [13.0, 16.9]	15.3 [13.1, 17.3]	0.074
Axial length, mm (mean ± SD)	23.4 ± 1.0	23.5 ± 1.0	0.621
**LOCS III grade, no. (%)**			
Grade 1	167 (44.4%)	1 (0.4%)	**< 0.001**
Grade 2	186 (49.5%)	48 (18.2%)	
Grade 3	23 (6.1%)	161 (61.0%)	
Grade 4	0 (0.0%)	54 (20.5%)	
**Drusen severity, no. (%)**			
None	112 (29.8%)	0 (0.0%)	**< 0.001**
Mild	195 (51.9%)	13 (4.9%)	
Moderate	65 (17.3%)	93 (35.2%)	
Severe	4 (1.1%)	158 (59.8%)	
Systemic comorbidities and biomarkers			
Hypertension, yes, no. (%)	250 (66.5%)	228 (86.4%)	**< 0.001**
Diabetes mellitus, yes, no. (%)	186 (49.5%)	145 (54.9%)	0.201
Total Cholesterol, mmol/L (mean ± SD)	4.98 ± 0.82	4.95 ± 0.75	0.682
LDL-C, mmol/L (mean ± SD)	2.51 ± 0.55	2.50 ± 0.51	0.759
C-reactive protein, mg/L (median [IQR])	1.44 [0.93, 2.12]	1.68 [1.16, 2.86]	**< 0.001**

SD, standard deviation; IQR, interquartile range; BMI, body mass index; BCVA, best-corrected visual acuity; IOP, intraocular pressure; AL, axial length; LOCS III, lens opacities classification system III; LDL-C, low-density lipoprotein cholesterol.

Continuous variables are presented as Mean ± SD or Median [IQR] depending on normality distribution. Categorical variables are presented as number (percentage). Bold values indicate statistical significance (*P* < 0.05).

Comparison of continuous clinical features revealed distinct pathophysiological patterns between the groups (Figure 1B). The age of the patients in the comorbidity group was significantly higher than that of the control group (mean 73.8 vs. 67.7 years, *P* < 0.001), confirming age as a primary risk factor. Additionally, the body mass index (BMI) and intraocular pressure (IOP) were also much higher in the comorbidity group than controls (median BMI: 25.3 vs. 24.1 kg/m^2^, *P* < 0.05), hence the possible involvement of the metabolic status and ocular dynamic in the pathogenesis of the disease. On the other hand, low-density lipoprotein cholesterol LDL-C levels did not differ significantly between the two groups (mean 2.50 vs. 2.51 mmol/L, *P* > 0.05), suggesting that LDL-C as an individual bio-predictor does not appear to be a discriminating biomarker in the given cohort.

In the case of categorical variables (Figure 1C), the comorbidity group contained significantly more smokers (33.0% vs. 19.1%, *P* < 0.001) and patients with hypertension (HTN) (86.4% vs. 66.5%, *P* < 0.001) than the control group had. Also, the cataract degree was significantly high in the comorbidity group with more patients having developed lens opacities in LOCS III categories 3 and 4 (81.5% vs. 6.1%, *P* < 0.001).

To determine relationships between features and how they can be separated, the exploratory data analysis was performed. Pairwise correlations between 15 clinical features showed low-to-moderate correlations (Figure 1D), and thus, it was only expected between biologically related variables (e.g., LOCS III and BCVA), which reduced the risk of the issue of multicollinearity. The visualization of the high-dimensional feature space (Figure 1E) was shown as the t-Distributed Stochastic Neighbor Embedding (t-SNE), which showed a complicated distribution. Although there were clear clustering patterns between the control condition (blue) and comorbidity (red) groups, the boundary was non-linear with substantial overlap, necessitating the use of complex machine learning algorithms rather than simple linear classifiers.

### 3.2 Performance comparison and model evaluation

**Figure 2:**
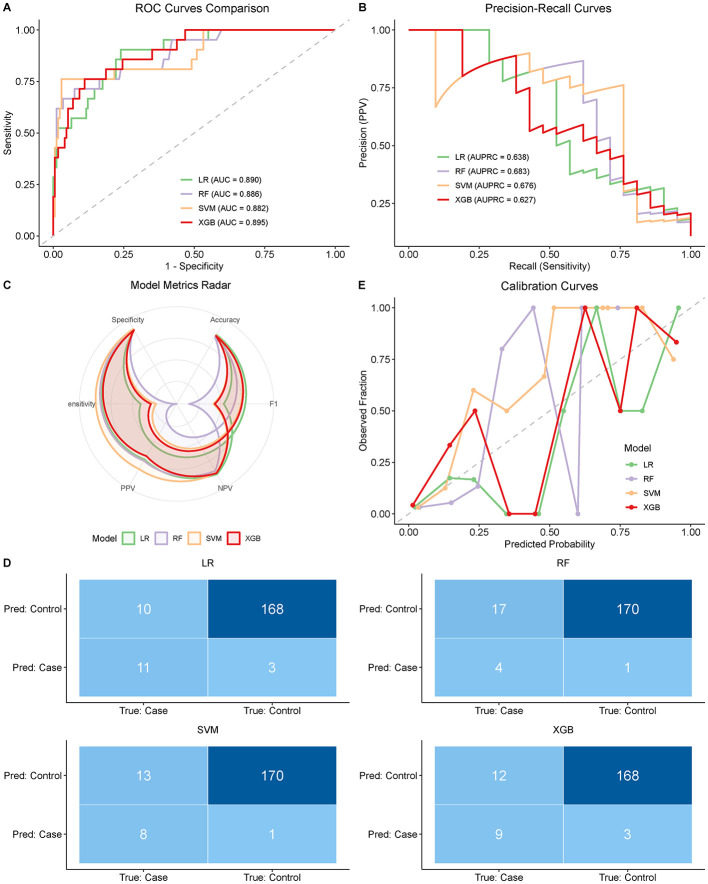
Performance evaluation and comparison of machine learning models. **(A)** Receiver Operating Characteristic (ROC) curves for Logistic Regression (LR), Random Forest (RF), Support Vector Machine (SVM), and XGBoost (XGB) models. The Area Under the Curve (AUC) values indicate model discrimination performance. **(B)** Precision-Recall Curves (PRC) illustrating model performance in identifying positive cases, with Area Under the Precision-Recall Curve (AUPRC) values reported. **(C)** Radar chart comparing multiple performance metrics (Accuracy, Sensitivity, Specificity, Positive Predictive Value [PPV], Negative Predictive Value [NPV], and F1-score) across the four models. **(D)** Confusion matrices for each model on the test set, showing the number of true positives, false positives, true negatives, and false negatives. **(E)** Calibration curves assessing the agreement between predicted probabilities and observed frequencies. Ideally, the curve should align with the diagonal dashed line.

To determine the best algorithm to use in predicting comorbidity risks, we compared four machine learning models, namely, Logistic Regression (LR), Random Forest (RF), Support Vector Machine (SVM), and XGBoost (XGB). The Receiver Operating Characteristic (ROC) curve was mostly used to discriminatively evaluate the performance of every model (Figure 2A). The XGBoost model demonstrated robust discriminative performance (AUC = 0.895, 95% CI: 0.85–0.93), comparable to Logistic Regression (AUC = 0.890), but offered unique advantages in visualizing non-linear risk factors. The performance of all the models was robust, and all of them had AUC values exceeding 0.88.

The dataset presented a relatively balanced distribution (prevalence of comorbidity = 41.25%, *n* = 264/640). To ensure consistent class distribution across subsets, stratified sampling was employed to partition the data into training (70%) and testing (30%) sets. Precision-Recall Curves (PRC) were additionally applied to assess model performance comprehensively (Figure 2B). The Area Under the Precision-Recall Curve (AUPRC) values were ranging from 0.627 (XGB) to 0.683 (RF). All the models demonstrated substantial improvement over the random baseline probability (0.109), which demonstrates a strong predictive power.

These detailed performance metrics are thoroughly compared in the radar chart (Figure 2C) and confusion matrices (Figure 2D). The highest specificity (0.994) was obtained with the Random Forest model but its clinical utility was limited by its remarkably low sensitivity (0.190), which gave the model a high false-negative rate (17 missed of 21 positive samples). Conversely, Logistic Regression, and XGBoost had a better performance profile. Logistic Regression produced the best sensitivity (0.524) and F1-score (0.629) with the same result (11-true positive). The XGBoost model was highly specific (0.982) and accurate (0.922) and moderately sensitive (0.429) and F1-score (0.545).

Besides, the agreement between the expected probabilities and the results was measured by the calibration curves (Figure 2E). The XGBoost and LR models provided a good fit to the ideal diagonal, which is an indication that the predicted risk scores are reliable estimates of the actual disease probability within this cohort. Based on the trade-off between sensitivity (maximum in LR) and discrimination (maximum in XGBoost) and the ability to model non-linear interactions, XGBoost was chosen as the prime model to be used in further interpretability analysis.

### 3.3 Global interpretability and feature importance analysis

**Figure 3:**
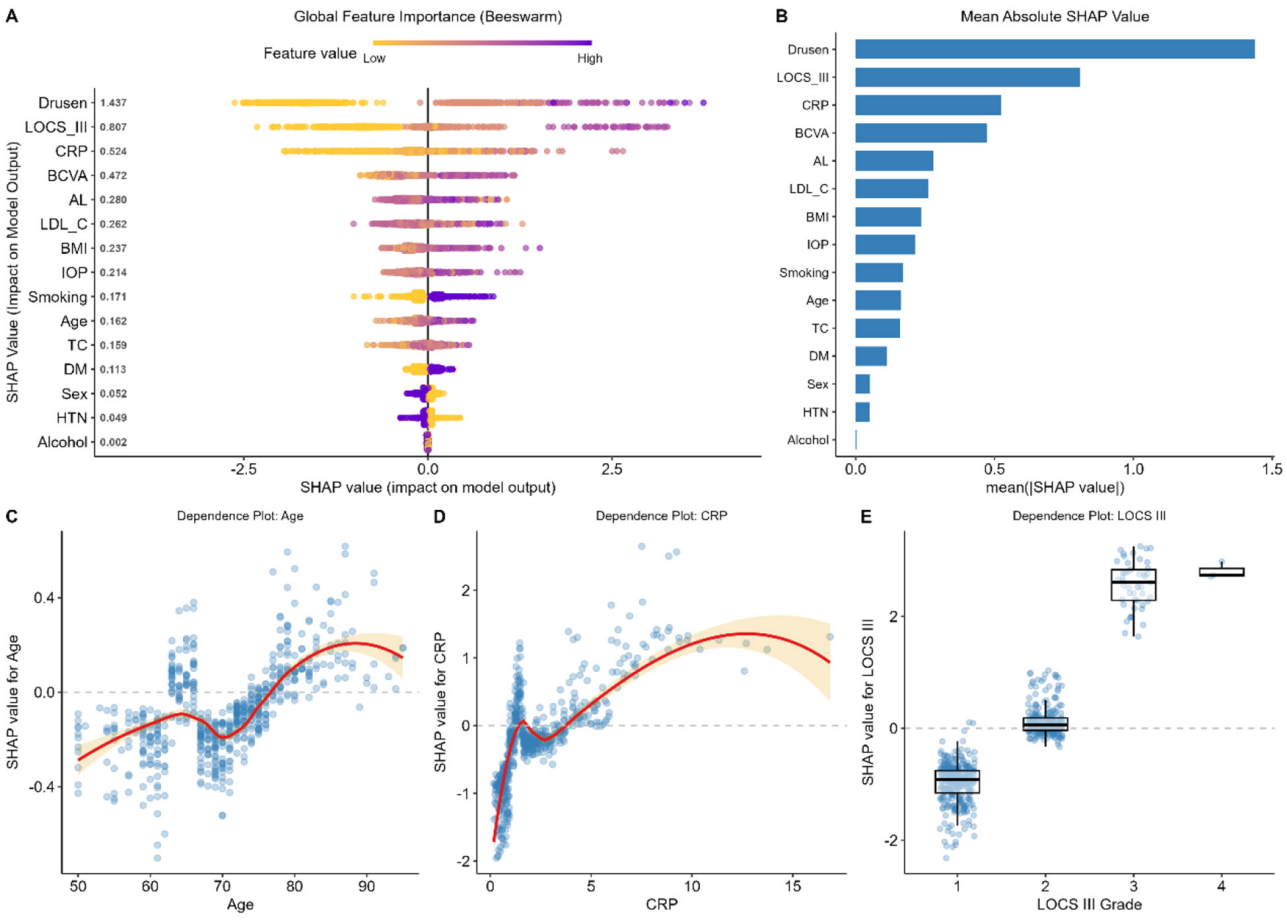
Global interpretability analysis using SHAP (SHapley Additive exPlanations). **(A)** SHAP summary beeswarm plot displaying the impact of the top 15 features on the model output. Each dot represents a patient; color indicates the feature value (red = high, blue = low). Points to the right of the zero line indicate a positive contribution to the predicted risk. **(B)** Bar chart showing the mean absolute SHAP values, ranking features by their global importance. **(C-E)** SHAP dependence plots illustrating the non-linear relationships between specific features and their SHAP values: **(C)** Age, showing a sharp risk increase after 65 years; **(D)** C-reactive protein (CRP), demonstrating a threshold effect; and **(E)** LOCS III grade, showing a stepwise increase in risk. The red lines represent smooth curves (LOESS) fitted to the data points.

We used SHapley Additive exPlanations (SHAP) to measure the overall importance and directional effects of each clinical feature in explaining the decision-making process of the XGBoost model. The SHAP summary plot (Figure 3A) is the most effective for visualizing the overall model behavior, and the dots represent patients, and the color gradient shows the feature value [red (high), blue (low)]. Features are ordered by their mean SHAP absolute values (Figure 3B), which are all the available features in the model, and can assess their overall contribution to the model output.

In line with clinical expectation, ocular signs were pointed out as the dominant predictors. The most prominent feature turned out to be Drusen (mean |SHAP value|) (1.437), and then LOCS III grade (mean 0.807). Figure 3A indicates that positive values of Drusen and LOCS III (red dots) were strongly associated with positive values of SHAP, and the increase in the predicted risk of comorbidity was significant. On the other hand, the risk score was considerably lower; the lack of such signs (blue dots).

There were also systemic factors that were of significant roles. C-reactive protein (CRP) was the third rank (mean |SHAP value| = 0.524), which presents the value of systemic inflammation on the pathogenesis of the disease. Other lifestyle and metabolic factors, such as LDL-C, BMI, and Smoking were also found to be the major contributors though with lower their scores of importance than ocular signs.

In addition, the SHAP dependence plots showed complicated non-linear correlations between continuous variables and disease risk. Figure 3C gives the influence of Age which is S-shaped in nature with a relatively low risk of development until the age of about 65 after which the risk increases sharply before leveling off at an age of about 80. Likewise, Figure 3D reveals a threshold effect of CRP with risk increasing at high rates as CRP rises between 0 and 5 mg/L implying that there is a saturating effect of inflammatory influence. In the ordinal variable LOCS III (Figure 3E), a definite progression could be noted: grades 1–2 were connected with the negative values of SHAP (indicating a protective effect), grades 3–4 were significantly increased to positive ones, suggesting that advanced cataract is a strong indicator of comorbidity in the given model.

### 3.4 Analysis of feature interactions and synergistic effects

**Figure 4:**
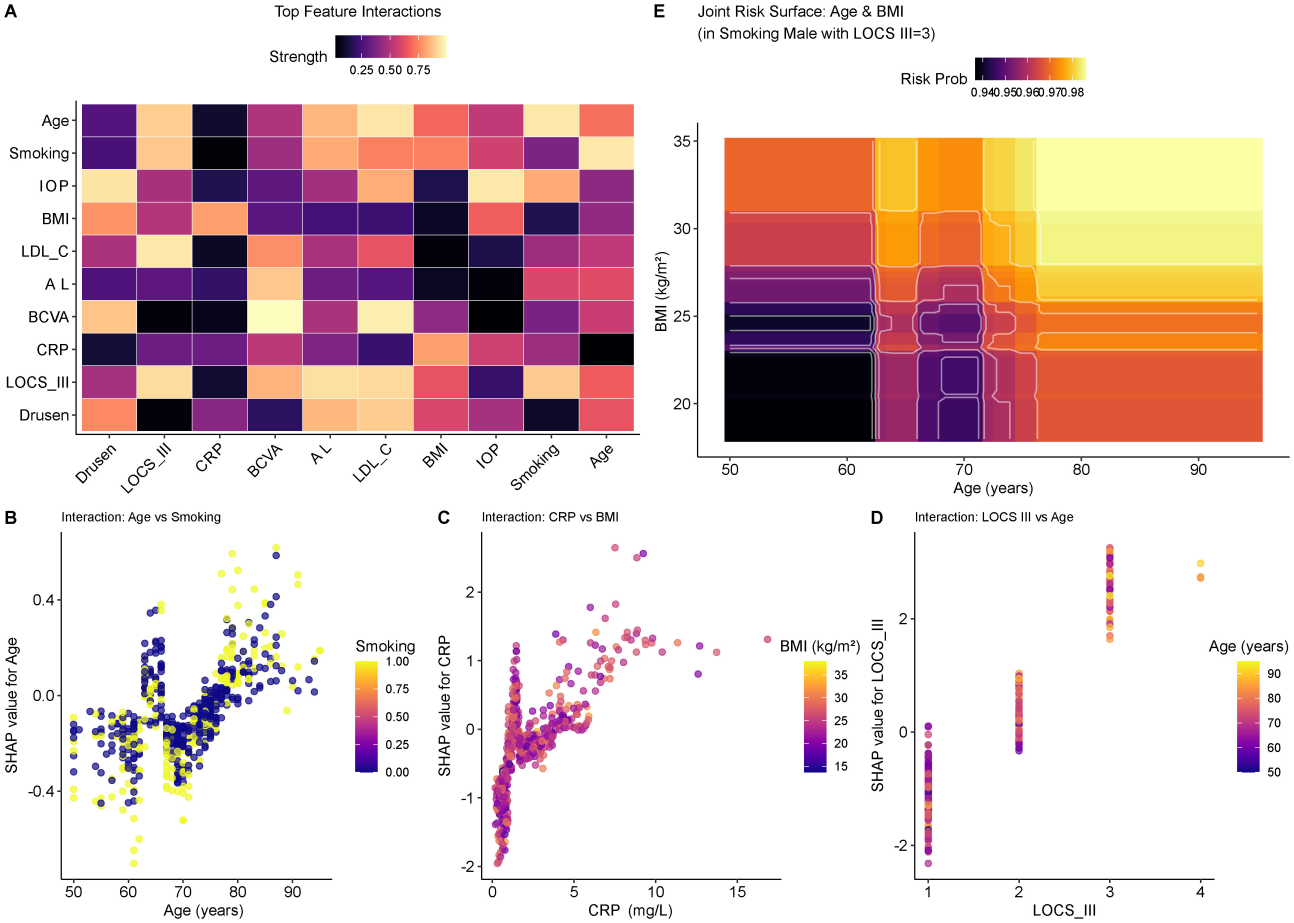
Analysis of feature interactions and synergistic effects. **(A)** Heatmap of top feature interactions, highlighting the strength of interactions between key variables. Lighter colors indicate stronger interactions. **(B-D)** SHAP interaction dependence plots revealing how the relationship between a primary feature and the SHAP value is modified by a second feature: **(B)** Age vs. Smoking, showing amplified risk in older adult smokers; **(C)** CRP vs. BMI; and **(D)** LOCS III vs. Age. Color scales represent the value of the interacting feature. **(E)** Joint risk surface plot visualizing the combined effect of Age and BMI on the predicted probability of comorbidity in a simulated high-risk profile (smoking male with LOCS III grade 3). Lighter colors (yellow/orange) indicate higher risk probabilities.

In order to exemplify the intricate nature of mutual interaction of risk factors, we computed feature interaction values with SHAP interaction values. Figure 4A of the heatmap of top feature interactions demonstrates the strongest pair-wise associations that lead to the prediction of the model. It is important to note that the correlation between Age and Smoking emerged as the most important one, followed by CRP and BMI, LOCS III and Age.

Figure 4B: The dependence plot of the AgeSmoking interaction shows that there is a pronounced synergistic effect. The SHAP values used in younger people (a 65 years old cutoff) are quite similar, which means there are similar and rather low-risk groups of smokers (yellow dots) and non-smokers (blue dots). However, with age, a clear divergence can be observed: the risk pathway of smokers developed exponentially, and that of non-smokers is much less steep. This distinct divergence is an indication that smoking is an age accelerant of the pathogenic processes, and it, as such, disproportionately increases the comorbidity risk of the older population (> 75 years).

Equally, the correlations of the systemic inflammation and the metabolic state were examined (Figure 4C). Scatter plot reveals that there is a positive correlation between the CRP and SHAP. Surprisingly, at elevated CRP concentrations (> 5 mg/L), persons with raised BMI (yellow/orange dots) are likely to be concentrated on the extreme right of the risk distribution than those with low BMI (purple/blue dots). This finding confirms the hypothesis that obesity can enhance the harmful impact of systemic inflammation on the state of the eyes.

Besides systemic factors, the dependence between ocular architecture and demographics was described by the use of the LOCS III vs. Age dependence plot (Figure 4D). Although the severity of the lens opacities was clearly the key to risk, such a vertical stratification driven by age was distinctively observed within each category of grades. Particularly, older individuals (symbolized by yellow dots) have shown much higher risk scores than their younger counterparts (purple dots) among the patients with the same level of lens clouding (e.g., LOCS III Grade 3). This result is an indication that chronological aging has a compounding effect on structural lens changes. It follows that pathological role of cataract concerning the presence of AMD has deeper pathological meaning in older adults, which is probably due to cumulative retinal senescence, present in addition to lenticular changes.

Additionally, joint risk surface plot (Figure 4E) is used to visualize the effect of both Age and BMI on the estimated probability of comorbidity on the prediction. In 2D contour map, the risks stratification is visually distinct: individuals in the upper-right quadrant (old age and high BMI) belong to the category of the highest predicted risk (probability exceeds 0.95, denoted by bright yellow), and the people on the lower-left parameter are in the safety range (dark blue). The non-linearity of the risk curves highlights why advanced machine learning models, like XGBoost, are required to be able to capture such complex and high dimensional dependencies that linear models may fail to capture.

### 3.5 Individualized prediction and local interpretability for representative cases

**Figure 5:**
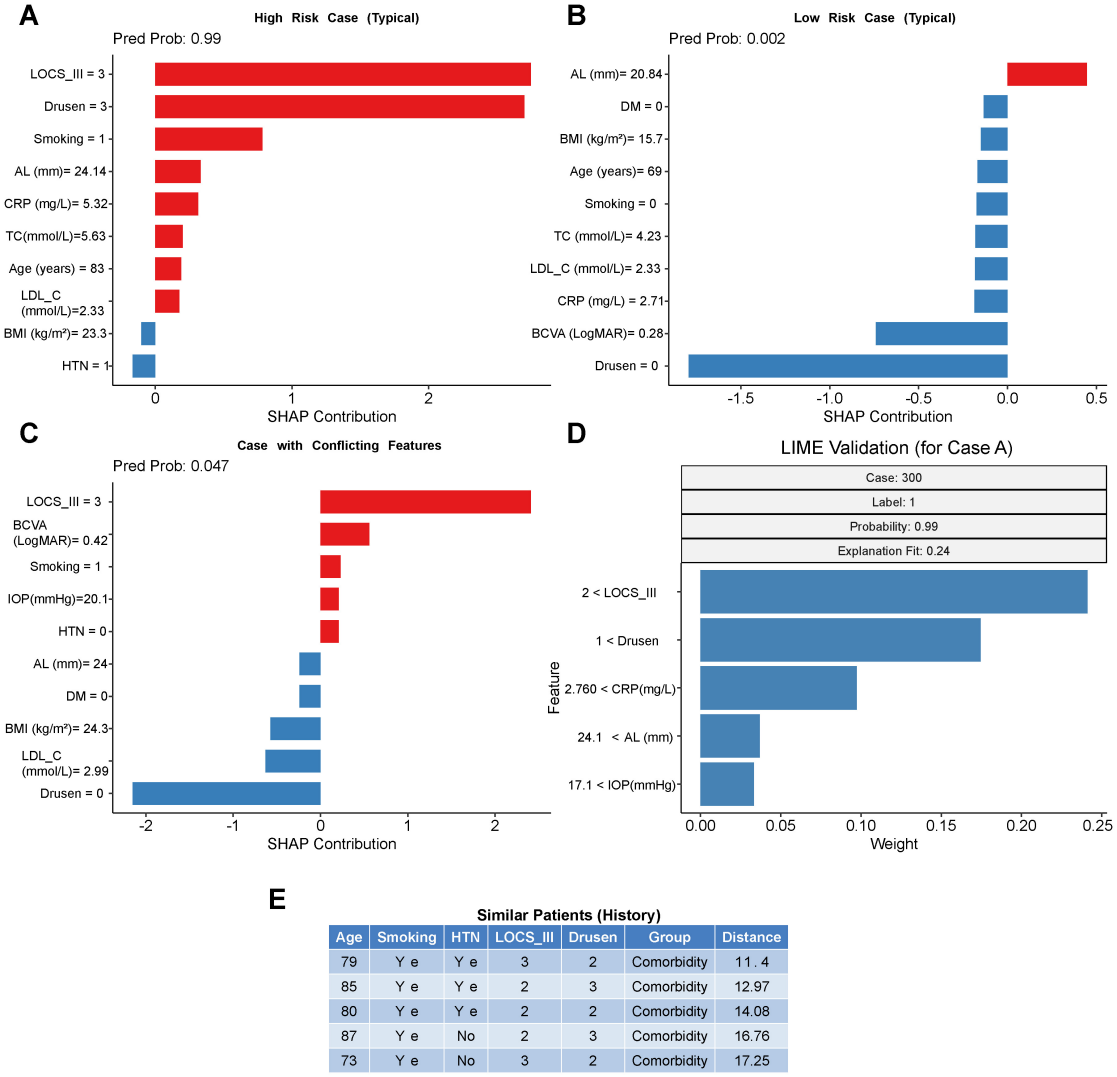
Individualized prediction and local interpretability for representative cases. **(A-C)** SHAP waterfall plots illustrating the contribution of each feature to the prediction score for specific individuals. Red bars indicate features increasing the risk, while blue bars indicate features decreasing the risk. **(A)** A correctly predicted high-risk case (True Positive). **(B)** A correctly predicted low-risk case (True Negative). **(C)** A case with conflicting features (high systemic risk but low ocular signs), demonstrating the model's decision process in complex scenarios. **(D)** LIME (Local Interpretable Model-agnostic Explanations) validation for Case A, corroborating the SHAP results. **(E)** Nearest neighbor analysis showing the clinical outcomes of the 5 patients in the training set most similar to the query case, providing evidence-based decision support.

In order to illustrate the clinical usefulness and transparency of the XGBoost model, we chose three representative cases, breaking them down to the local interpretability of the waterfall plots on SHAP (Figure 5A-C).

The typical comorbidity patient (predicted probability: 0.99) is seen in Case A (Figure 5A, High-Risk Profile). The model decision has mainly been influenced by the ocular findings with advanced grades of LOCS III (3 +2.75 SHAP value) and Drusen grade (3 +2.70 SHAP value) as they are the highest positive contributions to the risk score. The classification to high risk was strengthened by the systemic factors such as Smoking (+0.78) and high CRP (5.32 mg/L, +0.31), which complies with the already implicated multi-factorial etiology of the disease.

Figure 5B (Case B Low-Risk Profile) shows a control case (predicted probability: 0.002). No relevant pathological features (Drusen = 0,−1.79 SHAP value) and no status of smoker (Smoking = 0,−0.17 SHAP value) also had a strong impact on reducing the predicted risk although the patient was 69 years old. This case helps to point out the specifics of the model, which makes it impossible to eliminate comorbidity when there are no critical clinical symptoms, even in older adults.

The case C (Challenging Case with Conflicting Features, Figure 5C) presenting a complicated situation, the model successfully integrated conflicting clinical information (predicted probability: 0.047, which is correct, as a low-risk case). In spite of the fact that the patient exhibited significant risk factors of LOCS III grade 3(+2.41) and a history of Smoking, a negative influence (-2.15) outweighed the positive contributors, which was expressed by the retinal pathology (Drusen = 0). It shows that the model demonstrates potential utility in supporting differential diagnosis, to make a distinction between patients with isolated cataract and those with combined cataract and AMD, and not to add up the risk factors.

Moreover, the SHAP results were supported by the LIME analysis (Figure 5D) of Case A where the top predictors were explained as LOCS III, Drusen and CRP and, thus, proved model interpretability stability. Lastly, a case A (ID 300) was performed on a nearest neighbor analysis (Figure 5E). Five most similar patients found in the historical data were all referred to as comorbidity cases which is a good evidence-based support to the high-risk prediction of the model.

### 3.6 Clinical utility, robustness assessment, and translational potential

**Figure 6:**
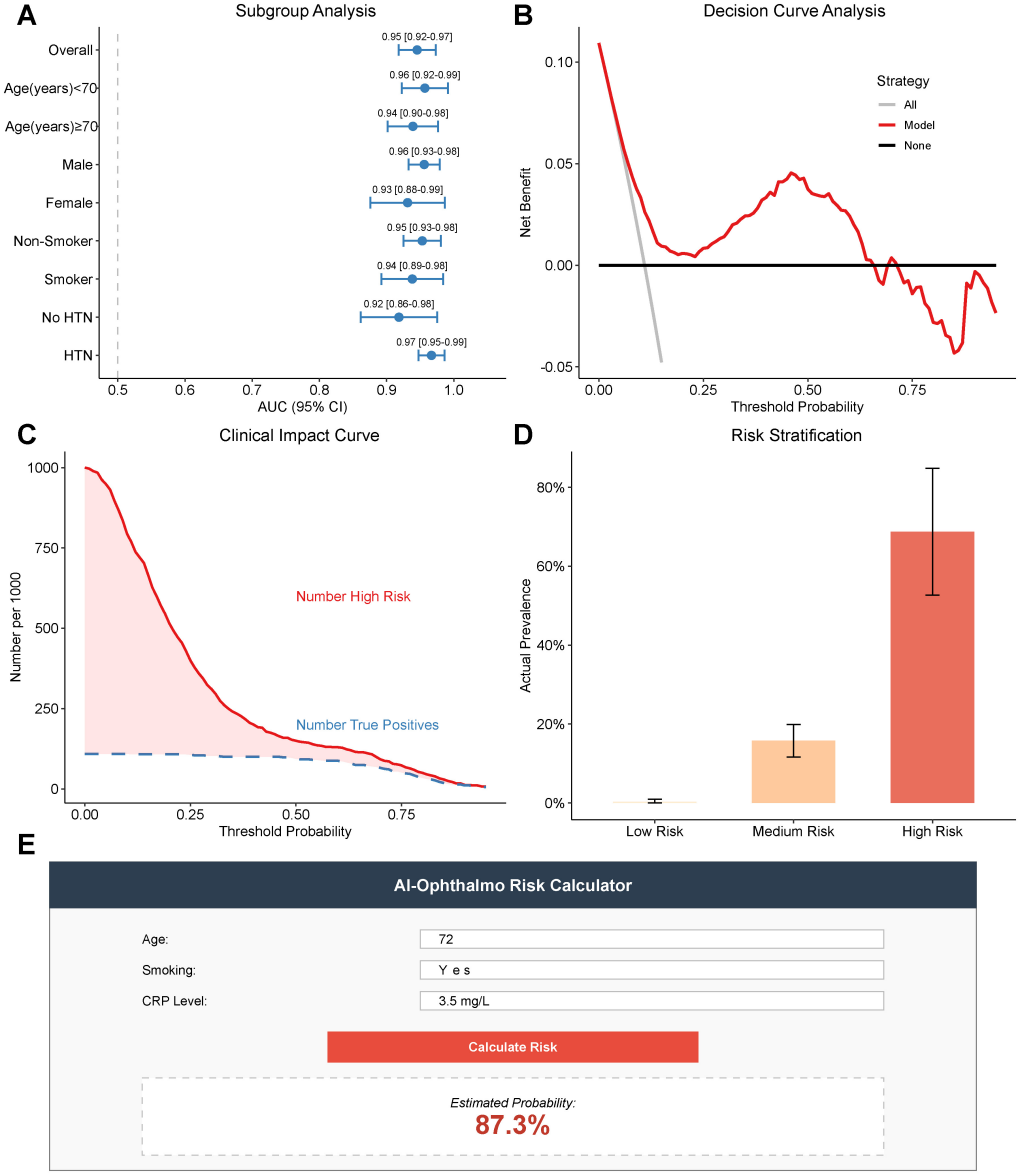
Assessment of clinical utility and model robustness. **(A)** Subgroup analysis forest plot showing the Area Under the Curve (AUC) with 95% confidence intervals across different demographic and clinical subgroups, demonstrating model stability. **(B)** Decision Curve Analysis (DCA). The red line represents the net benefit of using the model, which remains higher than the strategies of “treat all” (gray line) and “treat none” (black line) across a wide range of threshold probabilities. **(C)** Clinical impact curve comparing the number of people classified as high risk (red line) versus the number of true positive cases (blue dashed line) at different probability thresholds. **(D)** Risk stratification histogram showing the actual prevalence of comorbidity in patients stratified into Low (< 0.2), Medium (0.2–0.8), and High (>0.8) risk groups based on model predictions. Error bars represent standard errors. **(E)** Mockup of a web-based risk calculator, illustrating the potential for clinical translation.

To evaluate the reliability of the proposed model and the practicability of its application, comprehensive robustness and decision curve analyses were performed. As shown by subgroup analysis (Figure 6A), there have been uniform predictive performances in different patient strata. The model was very discriminative with an AUC of between 0.93 (Age ≥70 Years or older) and 0.96 (Age ≥70 Years or older) in the subgroups according to age (Age < 70, *n* = 300; Age ≥70, *n* = 340), gender (Male *n* = 326, Female *n* = 314), smoking (Smoker *n* = 237, Non-smoker *n* = 403), and hypertension. It is also important to note that despite the fact that the AUC is predictive in smokers (0.94) and non-smokers (0.95), the predictive power of the model does not rely on the primary risk factor of smoking but successfully incorporates other characteristics in proper classification.

To evaluate the net clinical benefit of the model, a Decision Curve Analysis (DCA) was used (Figure 6B). Our model curve (depicted in red) will always be located above the two extreme strategies which are denoted as: treat all (gray line) and treat none (black line) over a wide threshold probabilities range (say 0.10 to 0.85). This means that when the model is used in making screening or intervention decisions, the net benefit is high as compared to other strategies and there is no increment in the number of false positives. This is also supported by the Clinical Impact Curve (Figure 6C), which indicates that when the risk threshold is greater (above 0.4), the red line (high risk) and blue dashed line (true positive) are almost nearly equal to each other, which implies the positive predictive value is high, and the resource is efficiently distributed.

Figure 6D demonstrates the efficacy of the model of risk stratification. Predicted probabilities were used to classify the patients into low (< 0.2) and medium (0.2 0.8) risks and high-profile (>0.8) risks. The observed prevalence of comorbidity was steep in the high-risk group, which was 73.3% with a 0 step rate, whereas in medium-risk and low-risk, the prevalence was 12.2 and 0, respectively. This distinction demonstrates the potential of the model to stratify the patients well enough to comprehend them in such a way that they can have smaller clinical management approaches.

Lastly, to ease the application of these findings to clinical practice, we designed a prototype of a risk calculator web-based (Figure 6E). It is a cost-effective tool that only needs easily available clinical data (e.g., Age, Smoking, CRP) to output a personalized likelihood of cataract and AMD comorbidity, which may be a fast useful screening resource of ophthalmologists in hectic clinical practices.

## 4 Discussion

This study successfully developed and validated a machine learning framework to predict the risk of cataract and AMD comorbidity using multi-dimensional clinical data. Our XGBoost model achieved superior predictive performance (AUC = 0.895) compared to traditional statistical methods. Clinically, an AUC of 0.895 signifies that in nearly 90% of randomly selected cases, the model will correctly assign a higher risk score to a patient who actually develops the comorbidity than to a healthy control. This excellent discriminatory capacity suggests that the model can be a highly reliable triage tool in busy ophthalmology clinics, effectively minimizing both false alarms and missed diagnoses. In addition to proper prediction, SHAP and LIME methods were also used to unmask the black box of the algorithm, which detected glassy drusen and lens opacity severity (LOCS III) as the most important ocular predictors, and disaggregated the essential contributory effects of systemic inflammation (CRP) and smoking. The original result of the given study is the measurement of non-linear synergistic interaction between aging and smoking, which means that lifestyle modification can contribute to a massive shift in the pattern of disease even in the genetically-determined older adult groups.

Our findings align with seminal epidemiological evidence, such as the Beaver Dam Eye Study and the Blue Mountains Eye Study, which established age and smoking as foundational risk factors (25, 26). A key distinction of our study from earlier epidemiological research lies in the explicit quantification of non-linear interactions via XAI. While previous studies identified age and smoking as independent risks, our model reveals how these factors act synergistically, providing a more nuanced risk profile that linear models fail to capture. Although most conventional regression analyses in past cohorts modeling smoking have usually considered it as a risk factor that is independent and additive, in our SHAP interaction analysis (Figure 4B), we find that there is a non-linear relationship characterized as a synergy between smoking and risk factors (27, 28). We observed that the risk difference between smokers and non-smokers linearizes moderately at mid-life but exponentially at the age of 75 and above. This observation indicates that cumulative oxidative lesions caused by smoking will result in impaired homeostatic reserve of the eye in the aging microenvironment, which eludes the linear nature of the conventional statistical model deployed in previous research (29).

In the context of systemic inflammation, the relationship between CRP and ocular comorbidity has been debated and inconsistent relationships have been found (30). Our machine learning strategy offers a plausible data-driven explanation. In contrast to the past studies which used the linear relationship between the levels of CRP and the probability of contracting a disease, the use of SHAP dependence plot (Figure 3D) showed a distinct saturation point of about 5 mg/L (31). This means that the pathogenic effects of the inflammation could cease to increase at some level. This non-linear finding is in agreement with conflicting reports in the literature: those studies, which make use of populations with generally low CRP levels may experience a strong association, but studies with high inflammation levels at baseline levels may report weaker correlations (32, 33). Our model can generate a more refined insight into the phenomenon of the cataract and AMD-linking “inflammatory soil” hypothesis, by harvesting this threshold effect.

In Artificial Intelligence, recent innovations have mainly been on Deep Learning (CNN) models using fundus imaging alone (34, 35). These image based models achieve high diagnostic accuracy, but tend to be a black box, which cannot be interpreted clinically and does not include systemic metabolic data (36). In comparison, our XGBoost model combines a variety of clinical characteristics, including ocular biometry to serum biomarkers, to compete on an AUC of 0.895. Though the Logistic Regression was as well able to perform strongly (AUC = 0.890) in our internal validation because the presence of drusen is a strong feature to inform the model, it lacked the capacity to capture the complex, non-linear interactions revealed by our model. The specific novelty of our study lies in quantifying the saturation threshold of CRP and the synergistic risk amplification between smoking and aging, providing granular insights that go beyond standard risk factor identification found in existing models. Thus, our research contributes to the existing state of AI development by indicating that interpretable machine learning in terms of structured clinical data can be used to supplement image-related diagnostic solutions, and can provide the valuable insights on systemic risks management image-only models cannot.

The identification of specific key factors carries profound clinical significance for routine ophthalmologic care. The prominence of Drusen and LOCS III as the top predictors validates the necessity of rigorous fundus examinations even when severe cataracts are present. Clinically, identifying high systemic risk (such as elevated CRP and heavy smoking) in a patient scheduled for cataract surgery should alert the clinician to prioritize macular assessments (e.g., using OCT) prior to the operation (37, 38). This is crucial because surgical trauma could potentially exacerbate subclinical AMD in a highly inflammatory systemic environment (39). Thus, identifying these factors enables ophthalmologists to transition from reactive treatment to proactive, targeted screening and perioperative management.

Although these are encouraging findings, there are a number of limitations that should be mentioned. However, several limitations warrant acknowledgment. First, the study is retrospective and this can add to selection bias due to the clear difference in distribution of smoking status amongst groups. Second, we also used systemic metabolic indicators, but not genetic markers (e.g., CFH, ARMS2), which would also increase the quality of the models. Third, A major limitation of this study is the reliance on a single-center dataset with internal validation. While the web-based calculator demonstrates potential, it remains a prototype. Its generalizability and clinical safety must be rigorously verified through external validation in multi-ethnic, multi-center cohorts before it can be adopted in routine clinical practice. Future research directions should focus on three aspects: First, longitudinal cohort studies are required to validate the causal relationships and the CRP threshold effect identified in this cross-sectional analysis. Second, integrating genetic susceptibility markers (e.g., CFH, ARMS2) with our clinical model could further enhance predictive precision. Finally, deploying this risk calculator in diverse clinical settings will help evaluate its real-world utility in improving patient outcomes. Lastly, since it was a cross-sectional study, the associations that were found, especially that of CRP threshold, should be further investigated in longitudinal research and to establish causality.

In conclusion, this research proposed a strong, understandable, and a clinically applicable machine learning application in terms of risk stratification of cataract and AMD comorbidity. Our model, which incorporates ocular signs with systemic biomarkers and shows their non-linear interactions, is a useful evidence-based support system of the personalized ophthalmologic screening and management.

## 5 Conclusion

This study presents a machine learning framework for predicting the comorbidity risk of cataract and age-related macular degeneration. By integrating multi-dimensional clinical features, our XGBoost model demonstrated superior discrimination (AUC = 0.895) and calibration compared to traditional linear approaches. Crucially, interpretability analyses via SHAP and LIME not only reaffirmed the diagnostic dominance of ocular signs but also unveiled the non-linear synergistic effects of systemic risk factors—specifically, how smoking accelerates age-related pathogenic processes and the threshold impact of systemic inflammation. These findings challenge the oversimplified linear assumptions often seen in conventional epidemiology. Clinically, the proposed risk stratification system and web-based calculator offer practical tools for identifying high-risk individuals, thereby optimizing resource allocation and facilitating timely, personalized interventions. Future research should focus on validating this model in multi-ethnic longitudinal cohorts to further pave the way for precision ophthalmology.

## Data Availability

The raw data supporting the conclusions of this article will be made available by the authors, without undue reservation.
